# Efficacy of Fumonisin Esterase in Piglets as Animal Model for Fumonisin Detoxification in Humans: Pilot Study Comparing Intraoral to Intragastric Administration

**DOI:** 10.3390/toxins14020136

**Published:** 2022-02-11

**Authors:** Kaat Neckermann, Gunther Antonissen, Barbara Doupovec, Dian Schatzmayr, James Gathumbi, Véronique Delcenserie, Silvio Uhlig, Siska Croubels

**Affiliations:** 1 Department of Pathobiology, Pharmacology and Zoological Medicine, Faculty of Veterinary Medicine, Ghent University, Salisburylaan 133, 9820 Merelbeke, Belgium; kaat.neckermann@ugent.be (K.N.); gunther.antonissen@ugent.be (G.A.); 2 Department of Food Sciences and Fundamental and Applied Research for Animal Health (FARAH), Faculty of Veterinary Medicine, University of Liège, Avenue de Cureghem 10, 4000 Liège, Belgium; veronique.delcenserie@uliege.be; 3 BIOMIN Research Center, Technopark 1, 3430 Tulln, Austria; barbara.doupovec@dsm.com (B.D.); dian.schatzmayr@dsm.com (D.S.); 4 Department of Pathology, Parasitology and Microbiology, Faculty of Veterinary Medicine, University of Nairobi, P.O. Box 29053, Nairobi 00625, Kenya; jkgathumbi@uonbi.ac.ke; 5 Toxinology Research Group, Norwegian Veterinary Institute, P.O. Box 64, 1431 Ås, Norway; silvio.uhlig@vetinst.no

**Keywords:** biomarkers, detoxifier administration route, efficacy, fumonisin B1, fumonisin esterase, human model, mycotoxin, pig, Sa/So ratio, toxicokinetics

## Abstract

Fumonisins, a group of highly prevalent and toxic mycotoxins, are suspected to be causal agents of several diseases in animals and humans. In the animal feed industry, fumonisin esterase is used as feed additive to prevent mycotoxicosis caused by fumonisins. In humans, a popular dosage form for dietary supplements, with high patient acceptance for oral intake, is capsule ingestion. Thus, fumonisin esterase provided in a capsule could be an effective strategy against fumonisin intoxication in humans. To determine the efficacy of fumonisin esterase through capsule ingestion, two modes of application were compared using piglets in a small-scale preliminary study. The enzyme was administered intraorally (in-feed analogue) or intragastrically (capsule analogue), in combination with fumonisin B1 (FB1). Biomarkers for FB1 exposure; namely FB1, hydrolysed FB1 (HFB1) and partially hydrolysed forms (pHFB1a and pHFB1b), were measured both in serum and faeces using a validated liquid chromatography-tandem mass spectrometry (LC-MS/MS) method, and toxicokinetic parameters were calculated. Additionally, the serum sphinganine/sphingosine (Sa/So) ratio, a biomarker of effect, was determined using LC-MS/MS. A significantly higher Sa/So ratio was shown in the placebo group compared to both esterase treatments, demonstrating the efficacy of the esterase. Moreover, a significant decrease in serum FB1 area under the concentration-time curve (AUC) and an increase of faecal HFB1 AUC were observed after intraoral esterase administration. However, these effects were not observed with statistical significance after intragastric esterase administration with the current sample size.

## 1. Introduction

**Figure 1:**
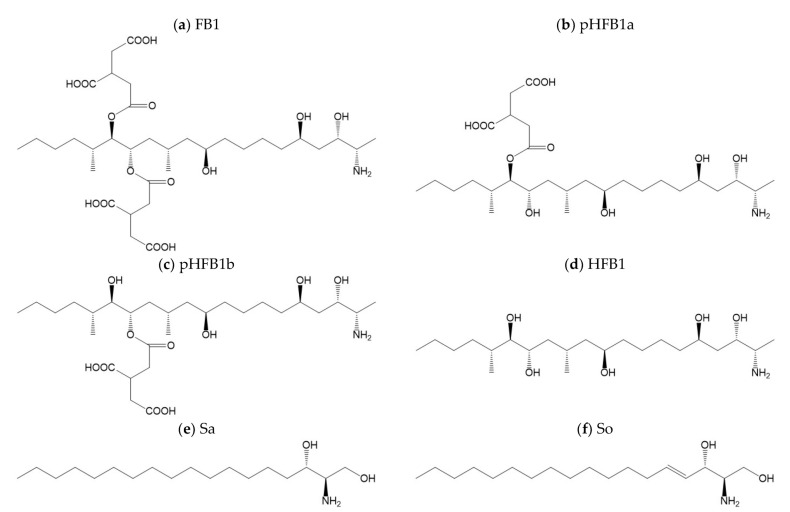
Structures of (**a**) fumonisin B1 (FB1), (**b**) partially hydrolysed FB1a (pHFB1a), (**c**) pHFB1b, (**d**) hydrolysed FB1 (HFB1 or aminopentol), (**e**) sphinganine (Sa) and (**f**) sphingosine (So).

Worldwide, 25% of food and feed is contaminated with toxic fungal metabolites with levels above the EU and Codex established limits [1]. However, the extent of the mycotoxin prevalence is underestimated as the aforementioned percentage neglects to take into account the amount of agricultural products that are contaminated with levels above detectable analytical levels, but below established limits [1]. The consumption of mycotoxin contaminated cereals, cereal derived products, or animal derived products is related to various types of diseases, or mycotoxicoses [2,3]. The disease can manifest itself acutely or chronically, such as the development of cancers and immune deficiency [2]. Of the more than 500 described mycotoxins, some are more prominent and/or more toxic compared to others and pose a greater risk to human and animal health. Fumonisins, produced by several species of *Fusarium* fungi, are one of the groups of mycotoxins with major importance; fumonisin B1 (FB1) being the most toxic and prevalent congener in nature (Figure 1) [3,4]. It is predominantly present in maize and maize-based products, which is the preferred staple food in most developing countries, especially in Latin America and Africa [5]. Fumonisin B1 has been classified as a group 2B ‘possibly carcinogenic to humans’ by the International Agency for Research on Cancer (IARC). In animals, FB1 intoxication has resulted in equine leukoencephalomalacia, a fatal brain disease in horses, porcine pulmonary oedema in pigs, neural tube defects in mice, and additionally hepatotoxic and nephrotoxic effects have been observed experimentally [6,7,8]. In humans, FB1 has been associated with an increased risk of oesophageal cancer, birth defects and adverse effects in liver and kidneys [8,9,10,11]. Furthermore, FB1 intake has been correlated to stunting in children [9,12,13] and has been shown to disrupt the proper functioning of the intestinal barrier [14]. Toxicity caused by FB1 and other fumonisins is plausibly linked to their inhibitory activity on sphinganine and sphingosine N-acyltransferase. Disruption of the sphingolipid biosynthesis results in a build-up of sphingoid bases and a decline of complex sphingolipids [14,15]. Sphingolipids are membrane lipids and play an important role in the regulation of fundamental cellular processes, i.e., cell division, differentiation, and apoptosis [16,17]. Hence, the sphinganine/sphingosine (Sa/So) ratio in blood or urine has been proposed as a reliable biomarker to evaluate fumonisin exposure and to demonstrate an adverse effect both after acute and chronic fumonisin exposure [18,19]. In animals, the Sa/So ratio in serum has been proven to be a reliable method to assess exposure [20]. In humans, the use of this ratio in urine or blood has been questioned [21]. Yet, the Sa/So ratio was noted to be useful when the FB1 contamination is high [22]. Despite the high level of variability even under controlled circumstances [23], the analysis of FB1 in urine is put forward as a valuable biomarker in humans [24]. Due to poor oral absorption, FB1 and its metabolites are predominantly excreted through the faeces (ranging from 52 to 94%) [25], making faecal sampling and analysis valuable. Furthermore, in addition to FB1 itself, its hydrolysed metabolites, i.e., partially hydrolysed FB1a (pHFB1a), pHFB1b, and hydrolysed FB1 (HFB1), were proposed to be useful as short-term biomarkers (Figure 1) [20].

To prevent acute and chronic mycotoxicosis in animals and humans, several pre- and postharvest intervention strategies are implemented [26,27]. Preharvest mitigation strategies include, among others, breeding of crops with enhanced resistance against disease and infection with mycotoxigenic fungi, and implementing optimal agricultural practices in the field [26,28]. Postharvest techniques include correct drying, sorting and shelling practices of maize, followed by proper storage and pest prevention [27]. Furthermore, some types of food processing, e.g., nixtamalisation and fermentation, have shown promising results [29,30]. Other mitigation strategies are the employment of detoxifiers as feed additives, namely mycotoxin binders or modifiers. Application of mycotoxin detoxifiers can decrease the amount of mycotoxins absorbed from the gut, thereby resulting in reduction or prevention of adverse health effects. The specificity of binders such as clay minerals is contested, although some tend to have a greater affinity for certain mycotoxins [31]. Modifiers change mycotoxin molecules into less toxic variants through conjugation with functional groups, ring cleavage, hydrolysis, deamination or decarboxylation [3,32]. Biological detoxifiers include application of certain microorganisms and their enzymes, i.e., bacteria, fungi, and yeasts; isolated from different sources, such as soil, animal gastrointestinal flora, and water. The advantage of enzymes for mycotoxin detoxification is their high specificity, [3]. One such enzyme for mycotoxin detoxification is an esterase, specifically designed to cleave the ester bonds in fumonisin side chains, releasing tricarballylic acid(s), and resulting in partially or fully hydrolysed FB1 (aminopentol) [33]. These metabolites have been demonstrated to be less toxic than the parent FB1 molecule [7,34,35]. In the human pharmaceutical industry, capsules as dosage form is widely adopted due to its many advantages. The capsule is self-administrative, odourless, tasteless, easy-to-swallow and can be manufactured in a variety of attractive colours [36]. Furthermore, it can be opened and its contents mixed in food. These characteristics would make a detoxifier enclosed in a capsule, an attractive form for human consumption.

In this study, a European Food Safety Authority (EFSA) approved feed additive, fumonisin esterase, was administered to piglets to compare the efficacy of two different administration routes, i.e., intraoral and intragastric [33]. In order to simulate the effect in humans as closely as possible, the pig was put forward as a suitable animal model. Pigs are physiologically and anatomically similar to humans regarding the gastrointestinal tract, liver, kidneys and cardiovascular organs [25,37]. The juvenile pig was used as model for the toddler; as young children are at a higher risk of mycotoxicoses due to higher exposure (a higher food intake/kg body weight (BW) and a higher consumption of cereal-based products), and a lower detoxification capacity due to metabolic and physiological immaturity [38,39,40]. In a previous experiment, this esterase demonstrated a promising effect in reducing FB1 concentrations in the human intestinal environment, using a toddler Simulator of the Human Intestinal Microbial Ecosystem [41]. A nearly complete reduction in FB1 concentration was achieved following addition of the enzyme.

The aim of this study was to investigate whether the FB1-degrading enzyme is able to reduce the exposure of FB1 in piglets through administration of the enzyme either intragastric (capsule imitation) or intraoral. The latter method of enzyme administration corresponds to the current employed standard route of in-feed administration of the commercial product. To the authors’ knowledge, no studies have been performed to investigate the possible efficacy of the enzyme when bypassing the mouth. The efficacy of the enzyme was determined by analysing relevant biomarkers for FB1 exposure and effect, and selected toxicokinetic parameters, and by comparing these parameters of the two administration routes to each other as well as to a placebo.

## 2. Results

To determine the effect of fumonisin esterase in relation to its route of administration, the biomarkers for FB1 exposure and effect in pigs listed in Table 1 were analysed in serum and faeces.

### 2.1. Biomarkers in Serum

**Figure 2:**
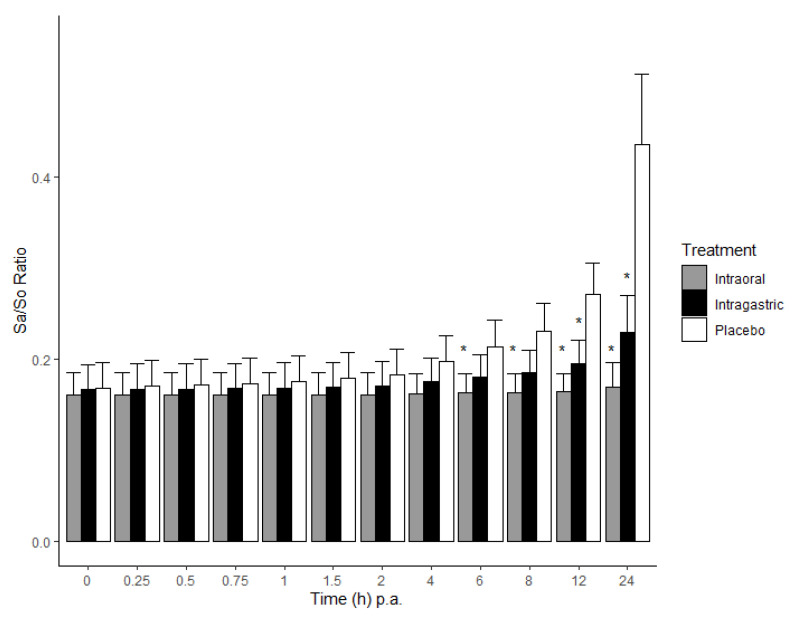
Mean sphinganine/sphingosine ratio (Sa/So) determined in pig serum during 24 h post-administration (p.a.) of a single intraoral administration of fumonisin B1 (2 mg/kg BW), either with a placebo (control group, *n* = 7), or with fumonisin esterase intraoral (*n* = 8) or intragastric (*n* = 7) administration. Error bars are the 95% confidence intervals. The asterisks represent a significant statistical difference (*p* < 0.05) compared to the placebo group.

Before the administration of FB1 to 22 piglets, the mean Sa/So ratio (±standard deviation, SD) was determined as 0.15 ± 0.02. Following administration of FB1, a significantly higher Sa/So ratio was observed in the placebo group compared to that of the intraoral and intragastric esterase treatment (*p* < 0.05) from 6 h and 12 h onwards, respectively (Figure 2). No significant difference was demonstrated between the intraoral and intragastric treatments. Throughout the 24 h trial, the Sa/So ratio in the intraoral treatment group remained stable. The highest mean Sa/So ratio was observed 24 h post treatments, and was 0.41 ± 0.09, 0.17 ± 0.03 and 0.23 ± 0.12 in the placebo, intraoral and intragastric groups, respectively. The observed increase in the Sa/So ratio can be attributed to an increase in Sa concentration rather than a decrease in So concentration.

**Figure 3:**
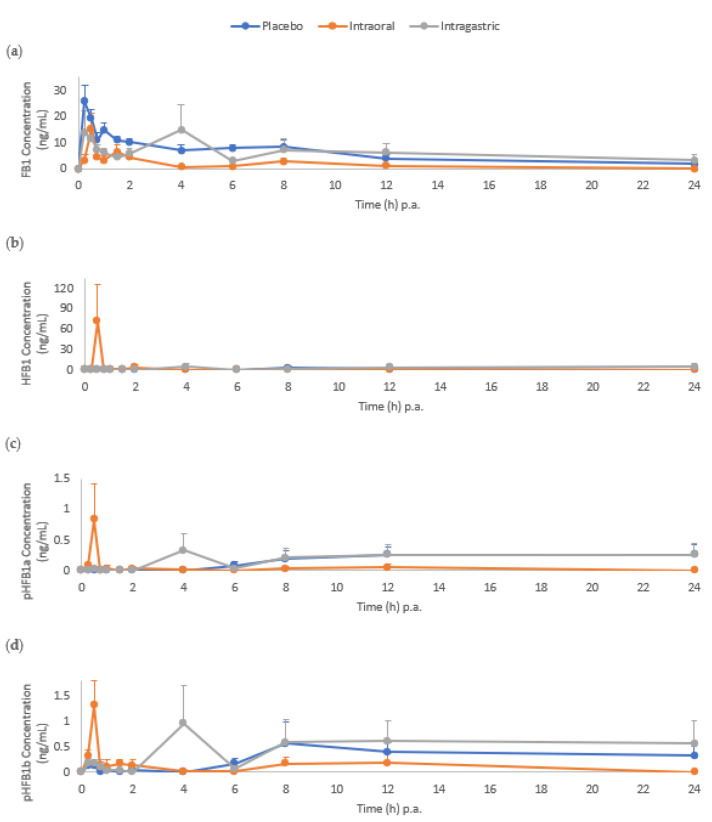
Mean concentration-time curve (+standard error of the mean, SEM) of (**a**) fumonisin B1 (FB1), (**b**) hydrolysed fumonisin B1 (HFB1), (**c**) partially hydrolysed fumonisin B1a (pHFB1a) and (**d**) pHFB1b, determined in pig serum after a single intraoral administration of FB1 (2 mg/kg BW), either with a placebo (control group, *n* = 7, blue curve), with fumonisin esterase intraoral (*n* = 8, orange curve) or intragastric (*n* = 7, gray curve) administration. The scale of the y-axis is different for individual plots.

Following FB1 administration to pigs, FB1 and pHFB1b were measured in serum as early as 15 min p.a. in all three treatment groups (Figure 3). Whereas HFB1 and pHFB1a were only measured as early as 15 min p.a. in the intraoral treatment group. Hydrolysed FB1 was observed at levels above LOQ at all sampling time points in the intraoral group from 15 min up to 24 h p.a., with a peak at 30 min. This was not the case in the intragastric group, where HFB1 was only detected at concentrations above the LOQ from 4 h p.a. and onwards. In the placebo group, it took up to 8 h p.a. to detect levels of HFB1.

An overview of the mean (± standard error of the mean, SEM) toxicokinetic parameters is presented in Table 2. A statistically significant (*p* < 0.01) decrease of 59.8% in the FB1 area under the concentration-time curve (AUC_0__→t_), from time zero p.a. to time of last measurable concentration, in the intraoral treatment group compared to the placebo was observed. On the contrary, this effect was not observed in the intragastric treatment group when compared to the placebo.

### 2.2. Biomarkers in Faeces

**Figure 4:**
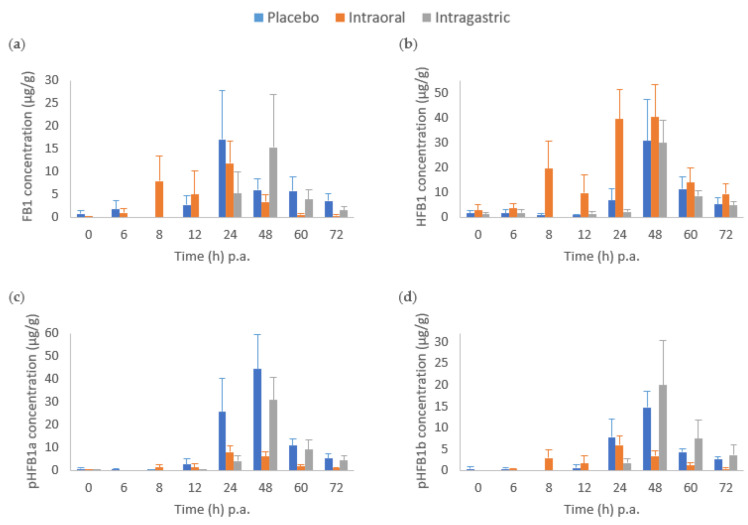
Mean concentration-time bar chart (+ standard error of the mean, SEM) of (**a**) fumonisin B1 (FB1), (**b**) hydrolysed fumonisin B1 (HFB1), (**c**) partially hydrolysed fumonisin B1 a (pHFB1a) and (**d**) pHFB1b, determined in pig faeces post-administration (p.a.) of a single intraoral administration of FB1 (2 mg/kg BW) either with a placebo (control group, *n* = 7, blue bar), or with fumonisin esterase intraoral (*n* = 8, orange bar) or intragastric (*n* = 7, gray bar) administration. The scale of the y-axis is different for individual plots.

Intragastric administration of fumonisin esterase resulted in a delayed FB1 peak (48 h p.a.), relative to placebo (Figure 4). Furthermore, there was a tendency to an earlier gradual increase in HFB1 concentration in the intraoral group compared to the other two treatment groups, while its concentration peaked at 48 h p.a. in both the placebo and intragastric groups (Figure 4). Similar to what was observed in serum, the (partially) hydrolysed metabolites of FB1 were also detected when no enzyme (placebo) was administered. Based on the toxicokinetic parameters determined in faeces, no statistically significant differences in the FB1 AUC from time zero to time of last measurable concentration at 72 h (AUC_0__→__t_) (h × µg/g) between the three treatments were observed (Table 3). However, for HFB1, a significant increase of 119% in AUC between the placebo and intraoral (*p* < 0.05) treatments was observed, as well as a significant difference in AUC between intraoral and intragastric (*p* < 0.05). Furthermore, a significant decrease in AUC of both pHFB1a (by 79.0%) and pHFB1b (by 60.0%) from placebo to the intraoral treatment was calculated. 

## 3. Discussion

The aim of this study was to determine the efficacy of fumonisin esterase for hydrolytic breakdown of FB1, when administered intragastrically and intraorally, and to compare it to placebo application. The rationale of the study was to imitate capsule ingestion, which is one of the most popular dosage forms on the market for pharmaceuticals and food supplements [36]. The evaluation of the efficacy was carried out by analysing relevant biomarkers of FB1 exposure and effect as reported in previous studies [42], as well as by investigating and comparing certain toxicokinetic parameters.

Fumonisin B1 disrupts the de novo synthesis of sphingolipids due to inhibition of the crucial ceramide synthase enzyme, resulting mainly in a build-up of Sa, causing a time-dependent increase in the Sa/So ratio [43,44]. This correlates to the findings in this study, where the increase of the ratio in the placebo group was attributed to an increase in Sa. While the Sa/So ratio in human serum and urine is a contested biomarker for FB1 exposure, most likely due to relatively low FB1 exposure levels, it has been shown to be reliable in animals, such as pigs, horses, rodents, rabbits, chickens, ducks, monkeys and trouts [6,23,45]. Furthermore, in humans, the range of normal Sa and So levels is large and levels within one individual vary over time [21]. Schwartz-Zimmermann et al. [20] acclaimed this biomarker in serum to be the most reliable when compared to other recognised biomarkers and matrices. Furthermore, the Sa/So ratio was suggested to be the preferred choice to measure enzyme efficacy for fumonisin detoxification. However, to assess the effectiveness of this specific detoxifier, it has been pointed out that either high fumonisin uptake levels, or long-term studies including a large number of animals are required. Our study confirms the Sa/So ratio to be a relevant biomarker for high acute FB1 exposure (2 mg FB1/kg BW), which was also concluded by Schwartz-Zimmermann et al. [20] in a pig trial, and similarly described in a human study performed by Qiu and Liu [22]. In our study, one pig from the intragastric-treatment group was excluded from the datasets. It had a Sa/So ratio of 0.56, which was 3.6 times higher than the average ratio (0.15 ± 0.02) found in the other 22 pigs. Moreover, Schertz et al. [18] reported an average Sa/So ratio between 0.10 and 0.15 at time point 0 h, which is similar to the average of the remaining 22 pigs. In our study we show that without the supplementation of the hydrolysing enzyme (placebo group), the Sa/So ratio significantly increased when compared to both groups that received the enzyme. No statistical difference in the ratio was observed between the groups that received the enzyme either intraorally or intragastrically. This indicates the efficacy of the enzyme, independent of the route of administration. However, a gradual increase (although not statistically significant; see Figure 2.) in this Sa/So ratio for the intragastric group towards 24 h p.a. is visible compared to the intraoral treatment. More information on the longer-term effect on the Sa/So ratio could have been obtained if serum was collected beyond 24 h p.a. Schertz et al. showed the continued increase of the Sa/So ratio beyond 24 h in the placebo group relative to intraoral fumonisin esterase treatment following a similar single-dose exposure with FB1 (at 2.47 mg/kg BW) [18]. While the intragastric administration of fumonisin esterase has so far not been tested, it is noteworthy that we achieved a measurable statistical difference in the Sa/So ratio from the placebo as early as 12 h post-treatment (6 h for the intraoral treatment). Although this indicates a faster effect of the enzyme when administered intraorally, compared to intragastric administration, the intragastric treatment clearly prevented a significant increase of the ratio. In a previous piglet feeding study by Masching et al. [19], fumonisin esterase was mixed in the feed and administered over 42 days (d). The feed contained 2 mg FB1/kg feed (roughly 0.84 mg/d, estimated based on a consumption of 50 g of feed per day). In their study, Masching et al. could not observe an effect of FB1 on the Sa/So ratio even after 14 d. A first significant difference between the FB1 and the enzyme administered groups was observed after 28 d. The Sa/So ratio significantly increased to 0.26 ± 0.08 on day 28 and further increased to 0.39 ± 0.02 on day 42. In contrast, in a study where the administered single FB1 dose (2.47 mg/kg BW) was slightly higher as in our study, a significant difference to placebo was only observed after 24 h [18]. This could indicate a better effect of the enzyme for acute exposure compared to exposure that is more chronic. In addition, there could also be an effect related to how the animals were exposed to FB1, i.e., either being mixed in the diet versus administered as culture material on an empty stomach. Previous studies have confirmed the uptake of FB1 being much more rapid in fasted animals [46,47].

The observed effect of the fumonisin esterase on the Sa/So ratio was not reflected in the concentrations of FB1 and its hydrolytic metabolites in the serum and faecal samples. There was no statistical significant difference in AUC for FB1 or FB1-metabolites between the placebo and intragastric groups. Nevertheless, a significant difference in the AUC between intraoral enzyme administration and placebo further confirmed the efficacy of the enzyme. A significant decrease in the AUC of FB1 of nearly 60% in the intraoral treatment compared to the placebo group was observed in serum. This reduction is less than the 90% reduction in AUC of FB1 in serum that was observed in a similar single-dose treatment study performed by Schertz et al. [48], using the same detoxifier. The administration method of both FB1 and the enzyme, as well as the feeding state of the animals were the main differences to our study. While FB1 and the enzyme were both administered intraorally to fasted pigs in this study, they were mixed in the basal diet of fed pigs, in the aforementioned study. To our knowledge, all previous studies evaluating the efficacy of fumonisin esterase were carried out following the instructions on the leaflet, namely mixing the enzymatic product in the feed. The manufacturer claims the enzyme to be activated in the saliva (combination of moisture and pH) as a result of chewing, and it is assumed to perform most of its activity in the mouth.

The conversion of FB1 to the completely hydrolysed product HFB1 as result of the enzyme activity has been observed in previous studies [19,20,48]. This was confirmed in our study in the faecal samples by a significant increase of nearly 120% in the AUC of HFB1 in the intraoral group compared to the placebo. Accordingly, serum HFB1 levels above LOQ were measured in all samples from the intraoral group. Additionally, a significant decrease in the partially hydrolysed forms of FB1 (pHFB1a and pHFB1b) in the faeces of the intraoral group relative to placebo was observed. These results concur with what was observed in other studies [20,48] where the fumonisin esterase has been shown to cleave both side chains of FB1, rather than only a single one. This results in a decrease in the partially hydrolysed metabolites in favour of an increase of the fully hydrolysed metabolite HFB1. The natural gastrointestinal degradation of FB1 into its partially hydrolysed metabolites attributed to microbial hydrolysis, was previously observed both in vitro [19,49] and in vivo [20,48,50] experiments. Such microbial hydrolysis was also detected in this study, as levels of pHFB1a and pHFB1b were measured in both the serum and faecal samples in the placebo group (no enzyme administered). Even though most studies report a predominant formation of partially hydrolysed metabolites [19,41], HFB1 was equally observed at low concentrations in the placebo group where exclusively FB1 was administered, suggesting further degradation of the partially hydrolysed forms, most likely due to activity of the bacterial microbiota [51]. Furthermore, these findings were reflected in the serum in our study, where HFB1 levels were also observed in the placebo group.

The toxicokinetic analysis showed a maximum serum FB1 level in the placebo group at 2.2 h (T_max_) p.a. The serum T_max_ for the hydrolytic metabolites was observed later, at 5.6 h, 18 h and 9.9 h p.a. for HFB1, pHFB1a and pHFB1b, respectively. Our observed FB1 serum T_max_ was comparable to what was observed by Prelusky et al. [52] and Dilkin et al. [53] in pigs, i.e., between 60 and 90 min and after 2 h, respectively. In their studies, FB1 was applied intragastrically with a single dose. In contrast, Dilkin et al. observed maximum FB1 excretion in faeces already between 8 and 24 h p.a. [53], while in our study this was detected between 24 and 48 h p.a. According to literature data, when pigs were non-fasted and FB1 was applied with the feed, the FB1 faecal peak occurred later, after 48 h [48]. Although the maximum FB1 concentration in serum was observed earlier in this study compared to that by Schertz et al. [48] (9.5 h p.a.), at least in part explained by the difference in fasted vs fed state, respectively, Schertz et al. also reported delayed maximum concentrations for the metabolites. The importance of the feeding status of the experimental animals on the oral absorption and faecal excretion of the toxin has previously been pointed out [48], and was confirmed in this study. Furthermore, the feeding status can also negatively affect the effect of the enzyme. Consumption of the enzyme in capsule form might prevent full contact to FB1 contaminated food present in the gastrointestinal tract. Therefore, the possibility exists that the enzyme would not be able to perform or reach its full detoxifying potential. Further research would also be needed regarding the optimal dose of the enzyme to reach its maximum effect.

In this study, we observed an early peak in FB1 serum metabolite concentrations (HFB1, pHFB1a and pHFB1b) following intraoral application of fumonisin esterase, compared to the intragastric group. These findings could indicate a delay in absorption. However, similar delayed maximum concentrations were observed in the faecal samples following intragastric application of the fumonisin esterase, possibly indicating a slowed activity of the enzyme when administered intragastrically compared to intraorally. The hydrolytic FB1 metabolite profile after intragastric administration was somewhat comparable to the placebo group, whereby more distal microbiota is responsible for the metabolite formation. The lack of statistical significance could be ascribed to the large inter-individual variability between the pigs. 

Considering the impact of FB1 on human health, including the higher vulnerability of children, the preliminary data obtained in this study in piglets, as animal model for humans, show that the consumption of fumonisin esterase mixed in the food might reduce FB1 exposure and hence prevent its deleterious effects. However, further testing in humans would be necessary to confirm this hypothesis. Likewise, to determine the efficacy of the intragastric administration of the enzyme, more research is necessary, preferably involving a larger sample size and a longer period of sampling (>24 h p.a. blood sampling). Furthermore, even though the Sa/So ratio in serum had been identified as the most reliable biomarker for confirming fumonisin esterase efficacy [20], it would be advised to continue analysing both several FB1 biomarkers for exposure and effect to determine the efficacy of fumonisin esterase. The analysis of a combination of several biomarkers seems to remain relevant for future FB1 detoxification studies.

## 4. Conclusions

From the preliminary data obtained in this study, the hydrolysis efficacy of fumonisin esterase to degrade FB1 into its less toxic metabolites HFB1, pHFB1a and pHFB1b was evident. A significant increase of the Sa/So ratio was prevented by both intragastric and intraoral administration of the fumonisin esterase enzyme when compared to the placebo group. However, the efficacy of the enzyme when administered intragastrically was not reflected in the FB1, HFB1, pHFB1a and pHFB1b levels in serum and faecal samples, while this was observed in the intraoral group. Based on these results, for human use of this enzyme, capsule ingestion cannot be recommended; it can be advised to thoroughly mix fumonisin esterase in the food prior to consumption.

## 5. Materials and Methods

### 5.1. Animals

Twenty-four 4-weeks old piglets (12 males, 12 females) were obtained from Ra-Se Genetics^®^ (Ooigem, Belgium). Upon arrival, the pigs were housed in groups of three in standard pig stables at the Faculty of Veterinary Medicine, Ghent University (Merelbeke, Belgium). Piglets received *ad libitum* access to water and feed (Biggistart Opti^®^ flour, AVEVE Lammens Filip, Massemen, Belgium), and were provided with varying stable enrichment (rubber and rope-like chew toys, balls and towels) throughout the entire trial. The starter feed was tested for possible contamination with mycotoxins prior to the start of the experiment by a validated multi-mycotoxin liquid chromatography-tandem mass spectrometry (LC-MS/MS) method at Primoris (Zwijnaarde, Belgium). The feed contained low levels (143 µg/kg) of deoxynivalenol, below the recommended guidance value of the EU (2006/576/EC) [54]. After an acclimatisation period of 6 and 8 days, half of the piglets were divided into 3 treatment groups, respectively. One male piglet was euthanised before the start of the trial due to lameness and arthritis in both hind legs and a fever, and the administration of nonsteroidal anti-inflammatory drugs had no effect. All animals were weighed daily until the day prior to treatment administration.

The piglet trial was assessed and approved (on 20 June 2019) by the Ethical Committee of the Faculty of Veterinary Medicine and the Faculty of Bioscience Engineering of Ghent University with case number EC2019-37.

### 5.2. Experimental Design

To ensure sobriety and possible interaction, 12 h prior to and up to 4 h p.a., the animals were fasted, and the piglets were separated individually by wooden boards in each pen. All animals (mean BW ± SD, 8.38 ± 0.70 kg) received a single intraoral dose of FB1 (2 mg/kg BW). The pigs were allocated to three different treatments (4 males, 4 females per group). In the placebo group, the pigs (*n* = 7 (3 males, 4 females), 8.56 ± 0.50 kg) received maltodextrin (300 mg/kg BW) both intraorally and intragastrically through gavage. The intraoral-treatment group (*n* = 8, 8.33 ± 1.00 kg) received fumonisin esterase (3 U/kg BW) intraorally and maltodextrin (300 mg/kg BW) through gavage (Table 4). The intragastric-treatment group (*n* = 8, 8.29 ± 0.51 kg) received fumonisin esterase (3 U/kg BW) through gavage and maltodextrin (300 mg/kg BW) intraorally (Table 4).

**Figure 5:**
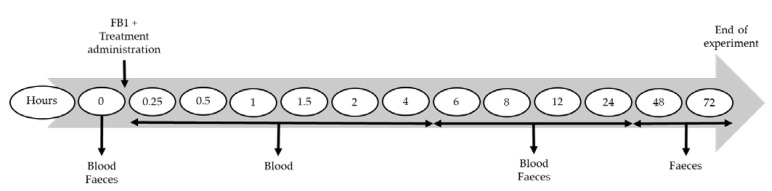
Sampling points in hours after fumonisin B1 (FB1) and treatment administration. Blood and faeces were collected at the different times indicated. Treatment consisted of fumonisin esterase administration intraorally or intragastrically, or maltodextrin (a placebo).

Eleven blood samples (2 mL each) per pig were collected in serum clot activator tubes (Vacutest Kima, Novolab, Geraardsbergen, Belgium) through venipuncture from the *vena jugularis* according to the time points presented in Figure 5. The collected serum tubes were placed upright and allowed to clot at room temperature during at least 30 min. The samples were centrifuged (10 min, 2851× *g*, 4 °C), and serum was stored at −20 °C until further analyses. All faeces were collected throughout the trial from the floor of each individual pen until 72 h p.a. Fresh faeces were collected at seven occasions (see Figure 5). These samples were lyophilised for approximately 48 h, ground (with mortar and pestle) and stored at −20 °C until further analysis.

In the majority of the piglets, salivation or gagging with small amounts of regurgitation was observed on average at 1.8 h following FB1 and treatment administration. Most likely, this was the result of aversion to the taste of FB1, which was provided as a culture material of *Fusarium verticillioides*. Due to fasting of the piglets and treatment administration in liquid form, passage through the stomach was deemed to have been mostly attained.

### 5.3. Products, Treatment Preparation and Administration

The FB1 culture material of *F. verticillioides* (containing 8.60 mg/g FB1) was obtained from Romer Labs (Tulln, Austria). The *F. verticillioides* strain M-3125 was cultured on rice, homogenised, and lyophilised [55,56]. Maltodextrin (placebo) and fumonisin esterase (10 U/g, FUM*zyme*^®^) were obtained from BIOMIN Holding GmbH (Tulln and Getzersdorf, Austria). The enzyme was initially identified and isolated from a soil bacterium *Sphingopyxis* sp. MTA144 [33,55,57]. The genes encoding the enzymatic activity were used to transform the yeast *Komagataella pastoris* into a fumonisin esterase secreting recombinant strain (*K. pastoris* DSM 26643) [33,55]. Maltodextrin was used as a carrier to produce the final product FUM*zyme*^®^.

All piglets received the FB1 culture material in powder form directly administered into the mouth. For the intraoral administrations of maltodextrin and fumonisin esterase, the products were left in powder form and administered, after homogenisation, via a 15 mL falcon tube (VWR, Leuven, Belgium) into the mouth. The mouth was held closed until swallowing occurred. For the intragastric administrations, the powders were dissolved in 20 mL of water and mixed vigorously, prior to intragastric administration with the help of a gavage tube.

### 5.4. Biomarker Analysis

Analysis of the Sa/So ratio in serum was carried out as described previously by Schwartz-Zimmermann et al. [20]. Serum aliquots (200 µL) were shaken with 600 µL of methanol/acetonitrile (50/50, *v*/*v*) for 30 min, followed by centrifugation at 14,000× *g*. Pellet extraction was performed with 300 µL of methanol/water (80/20, *v*/*v*), followed by centrifugation. The supernatant was dried and the residue reconstituted in 300 µL of acetonitrile/water (30/70, *v*/*v*). The solutions were centrifuged prior to LC-MS/MS analysis. The limit of quantification (LOQ) of Sa and So is 1.5 ng/mL in serum.

Quantification of FB1 and its metabolites in serum was carried out as previously described by Schertz et al. [48]. A ^13^C-labelled internal standard of FB1 was added to the thawed serum aliquots (300 µL), followed by the addition of 900 µL of methanol/acetonitrile (50/50, *v*/*v*). Samples were shaken (30 min, room temperature) and centrifuged (2800× *g*). Extraction of the pellets was performed twice with 200 µL of acetonitrile/water/formic acid (50/49/1, *v*/*v*/*v*), followed by centrifugation prior to analysis of the supernatant. The LOQs for FB1, HFB1, pHFB1a and pHFB1b analysis in serum are 0.39, 0.67, 0.14 and 0.21 ng/mL, respectively.

Fumonisin B1 and its metabolites, HFB1, pHFB1a and pHFB1b, were analysed in faeces as specified by Schwartz-Zimmermann et al. [20]. Briefly, extraction of the freeze-dried faeces aliquots (300 µg) was performed three times with acetonitrile/water/formic acid (74/25/1, *v*/*v*/*v*). The samples were centrifuged at 14,000× *g*, and the supernatant diluted 1 + 1 (v + v) with acetonitrile/water (30/70, *v*/*v*) prior to LC-MS/MS analysis. The LOQs are 0.74, 0.70, 0.81 and 1.0 µg/g for FB1, HFB1, pHFB1a and pHFB1b, respectively. 

All serum and faecal concentrations below the LOQ were excluded for the toxicokinetic and statistical analysis. 

### 5.5. Toxicokinetic and Statistical Analysis

One pig from the intragastric-treatment group was excluded from all datasets in serum and faeces, as its serum Sa/So ratio was abnormally high before the start of the treatment. Additionally, the high Sa/So ratio for this individual was identified as outlier at different time points using a Q-Q plot, and subsequently confirmed with a Bonferroni outlier test.

For statistical analysis of the Sa/So ratio in serum, a linear mixed effects (lme) model (with treatments as fixed effects and pigs as random effects) was applied using the software package RStudio [58]. Due to inherent non-linearity in the data, the prerequisite of residuals being normally distributed was not met, and therefore, the data were log transformed (log(Ratio)~Treatment*Time) to meet this criterium. 

Data obtained from the serum and faecal samples were analysed with regard to the following toxicokinetic parameters using non-compartmental analysis (Phoenix, version 8.1, Princeton, NJ, USA); maximum observed concentration (C_max_), time where maximum concentration was observed (T_max_), area under the concentration-time curve from time zero to time of last measurable concentration (AUC_0__→t_). The AUC was calculated with the linear-up/log-down trapezoidal method. The AUC was used to determine the efficacy of fumonisin esterase by comparing the two routes of administration (intraoral and intragastric) to placebo.

The detoxifier was considered effective when the AUC of FB1 for the control group (placebo) was significantly higher than the AUC of FB1 following esterase treatment. Furthermore, the detoxifier was equally regarded effective when the AUC of the control group (placebo) was significantly lower than the HFB1 AUC following esterase treatment.

The effect of the treatment, based on the AUC, was calculated as follows, and expressed as percent:

(1) $$\frac{\mathrm{Treatment}\;\mathrm{AUC}-\mathrm{Control}\;\mathrm{AUC}}{\mathrm{Control}\;\mathrm{AUC}}\times 100\;\left[\%\right]$$

Statistical analysis was performed in RStudio with a two-sided t-test with the level of significance set at 0.05.

## Figures and Tables

**Table 1:** Biomarkers for fumonisin B1 (FB1) exposure and effect measured in serum and faeces after administration of FB1 and fumonisin esterase to pigs.

Serum	Faeces
Sa/So ratio FB1 HFB1, pHFB1a, pHFB1b	FB1 HFB1, pHFB1a, pHFB1b
Sa/So (sphinganine/sphingosine) ratio, FB1 (fumonisin B1), HFB1 (hydrolysed fumonisin B1 or aminopentol), pHFB1a and pHFB1b (partially hydrolysed fumonisin B1a and B1b)	

**Table 2:** Mean ± standard error of the mean (SEM) of toxicokinetic parameters for fumonisin B1 (FB1) and its hydrolysed variants determined in serum after single oral administration of FB1 (2 mg/kg BW) to pigs, combined with either a placebo (control group, *n* = 7), or fumonisin esterase administered intraorally (*n* = 8) or intragastrically (*n* = 7).

Mycotoxin	Treatment	Maximum Observed Concentration (C_max_) (ng/mL) ± SEM	Time of Observed Maximum Concentration (T_max_) (h) ± SEM	Area under the Concentration-Time Curve Time Zero to Time Last Measurable Concentration (AUC_0__→t_) (h × ng/mL) ± SEM	Difference in AUC_0__→t_ between Control (Placebo) and Treated (Intraoral or Intragastric) Groups in %
FB1	Placebo	23 ± 3.8	2.2 ± 1.1	128 ± 15.5	
	Intraoral	10 ± 2.8	1.9 ± 0.9	51.4 ± 17.0 **	−59.8 **
	Intragastric	22 ± 7.8	7.0 ± 3.1	148 ± 51.7	+15.6
HFB1	Placebo	6.4 ± 4.8	5.6 ± 2.7	36.5 ± 31.0	
	Intraoral	33 ± 21	3.2 ± 1.2	49.8 ± 24.3	+36.4
	Intragastric	23 ± 6.9	11 ± 4.7	141 ± 54.4	+286
pHFB1a	Placebo	0.7 ± 0.2	18 ± 3.5	6.80 ± 2.18	
	Intraoral	0.6 ± 0.3	2.3 ± 1.5	2.12 ± 1.04	−68.8
	Intragastric	0.8 ± 0.3	13 ± 3.7	7.33 ± 2.82	+7.79
pHFB1b	Placebo	0.9 ± 0.4	9.9 ± 4.0	7.60 ± 2.43	
	Intraoral	0.9 ± 0.2	2.5 ± 1.2	3.07 ± 1.14	−59.6
	Intragastric	1.8 ± 0.7	11 ± 3.4	15.7 ± 6.60	+107

** *p* < 0.01, significantly different from placebo.

**Table 3:** Mean ± standard error of the mean (SEM) of toxicokinetic parameters for fumonisin B1 (FB1) and its hydrolysed variants determined in faeces after single oral administration of FB1 (2 mg/kg BW) to pigs, combined with either a placebo (control group, *n* = 7), or fumonisin esterase administered intraorally (*n* = 8) or intragastrically (*n* = 7).

Mycotoxin	Treatment	Maximum Observed Concentration (C_max_) (µg/g) ± SEM	Time of Observed Maximum Concentration (T_max_) (h) ± SEM	Area under the Concentration-Time Curve Time Zero to Time Last Measurable Concentration (AUC_0__→t_) (h × µg/g) ± SEM	Difference in AUC_0__→t_ between Control (Placebo) and Treated (Intraoral or Intragastric) Groups in %
FB1	Placebo	20 ± 10	39 ± 5.7	617 ± 305	
	Intraoral	16 ± 4.5	31 ± 4.4	391 ± 125	−36.6
	Intragastric	18 ± 13	52 ± 2.5	531 ± 384	−13.9
HFB1	Placebo	28 ± 15	33 ± 7.7	795 ± 323	
	Intraoral	66 ± 11	34 ± 5.6	1745 ± 289 *	+119 *
	Intragastric	30 ± 9.2	48 ± 0.0	796 ± 185 ^∆^	+0.13 ^∆^
pHFB1a	Placebo	56 ± 15	34 ± 4.8	1512 ± 333	
	Intraoral	11 ± 2.1	38 ± 5.3	318 ± 73.0 *	−79.0 *
	Intragastric	31 ± 9.9	48 ± 0.0	977 ± 335 ^a^	−35.4 ^a^
pHFB1b	Placebo	17 ± 3.9	38 ± 4.8	552 ± 109	
	Intraoral	8.2 ± 1.7	29 ± 6.0	221 ± 43.8 *	−60.0 *
	Intragastric	23 ± 12	48 ± 0.0	777 ± 420	+40.8

* *p* < 0.05, significantly different from placebo; ^∆^ *p* < 0.05, significantly different from intraoral; ^a^ *p* < 0.1, trend towards a intraoral-intragastric difference.

**Table 4:** Overview of the administered products, doses per kg body weight (BW) and the administration route per treatment group.

Treatment Groups	Products Administered	Dose (/kg BW)	Administration Route
Placebo (*n* = 7)	Fumonisin B1	2 mg	Intraoral
	Fumonisin esterase	-	-
	Maltodextrin	300 mg	Intraoral + Intragastric
Intraoral (*n* = 8)	Fumonisin B1	2 mg	Intraoral
	Fumonisin esterase	3 U	Intraoral
	Maltodextrin	300 mg	Intragastric
Intragastric (*n* = 8)	Fumonisin B1	2 mg	Intraoral
	Fumonisin esterase	3 U	Intragastric
	Maltodextrin	300 mg	Intraoral

## Data Availability

The data supporting the reported results of this study are included within the article.
